# The biological function of m6A demethylase ALKBH5 and its role in human disease

**DOI:** 10.1186/s12935-020-01450-1

**Published:** 2020-07-28

**Authors:** Jinyan Wang, Jinqiu Wang, Quan Gu, Yajun Ma, Yan Yang, Jing Zhu, Quan’an Zhang

**Affiliations:** 1 Department of Oncology, Nanjing Jiangning Hospital, The Affiliated Jiangning Hospital of Nanjing Medical University, Nanjing, 210000 China; 2 Department of Oncology, The Affiliated Jiangning Hospital of Jiangsu Health Vocational College, Nanjing, 210000 China; 3 Department of Oncology, Dafeng People’s Hospital, Yancheng, 224000 China; 4 Department of Oncology, The Affiliated Cancer Hospital of Nanjing Medical University, Nanjing, 210000 China

**Keywords:** ALKBH5, m6A, Cancer, Non-cancer, Mechanisms, Prognosis

## Abstract

Human AlkB homolog H5 (ALKBH5) is a primary m6A demethylase, which is dysregulated and acts as a biological and pharmacological role in human cancers or non-cancers. ALKBH5 plays a dual role in various cancers through regulating kinds of biological processes, such as proliferation, migration, invasion, metastasis and tumor growth. In addition, it takes a great part in human non-cancer, including reproductive system diseases. The underlying regulatory mechanisms of ALKBH5 that relys on m6A-dependent modification are implicated with long non-coding RNA, cancer stem cell, autophagy and hypoxia. ALKBH5 is also an independent prognostic indicator in various cancers. In this review, we summarized the current evidence on ALKBH5 in diverse human cancers or non-cancers and its potential as a prognostic target.

## Background

Chemical modifications of nucleobases were crucial for inducing changes in protein translation and regulating certain signaling pathways, which subsequently modulated kinds of biological processes. N6-methyladenosine (m6A) modification was first identified in mRNA-enriched RNA fractions in 1974, without any special attention [1]. m6A refered to methylation of the N6 position of adenosine base, which was one of the most prevalent and abundant internal modification in mammalian mRNAs and eukaryotes [2]. In recent years, with the application of high-throughput sequencing for detecting m6A, the understanding of the inside regulatory mechanisms came to light. m6A modification had been proved to be enriched in near stop codon and 3′untranslated terminal region (UTR) and translate near 5′UTR in a cap-independent manner [3]. m6A RNA modification of various RNAs, including messenger RNAs (mRNAs) [4], microRNAs (miRNAs) [5] and long non-coding RNAs (lncRNAs) [6], was a dynamic and reversible posttranscriptional modification process maintained by three different types of protein complex, including m6A readers, writers, and erasers [7]. This dynamic and reversible nature of the m6A modification made it pivotal in the rapid cellular communication. Recently, substantial progresses had been made in understanding m6A modifications in RNA metabolism, mRNA stability and splicing, translation efficiency, nuclear export, as well as alternative polyadenylation [8, 9].

**Fig. 1:**
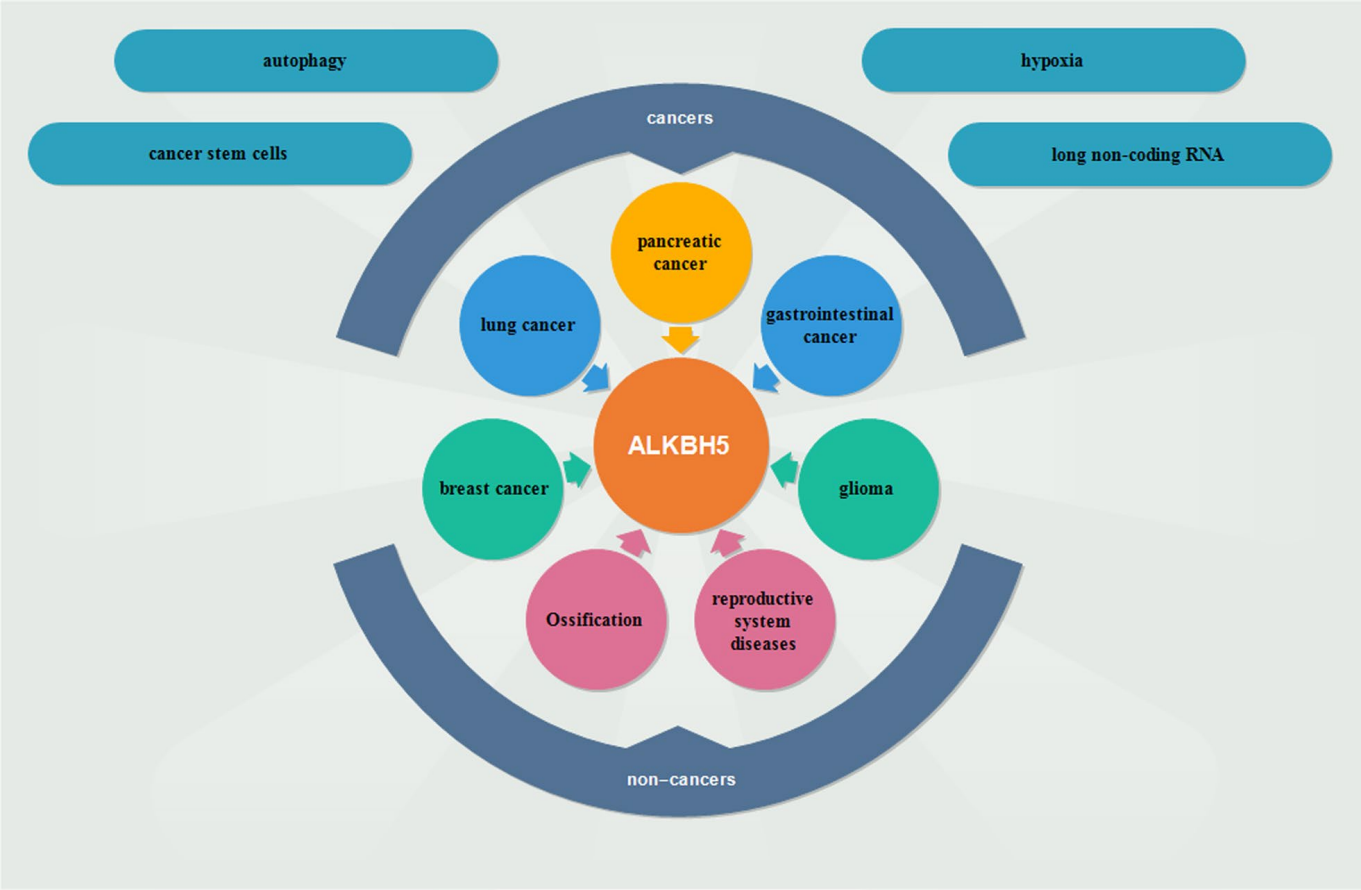
The mechanisms of ALKBH5 are involved in human cancers and non-cancers. ALKBH5 plays an important role in breast cancer, lung cancer, pancreatic cancer, gastrointestinal cancer and glioma. In addition, ALKBH5 takes a great part in human non-cancers, such as reproductive system diseases and ossification. The underlying mechanisms involve cancer stem cells, autophagy, hypoxia, long non-coding RNA

**Fig. 2:**
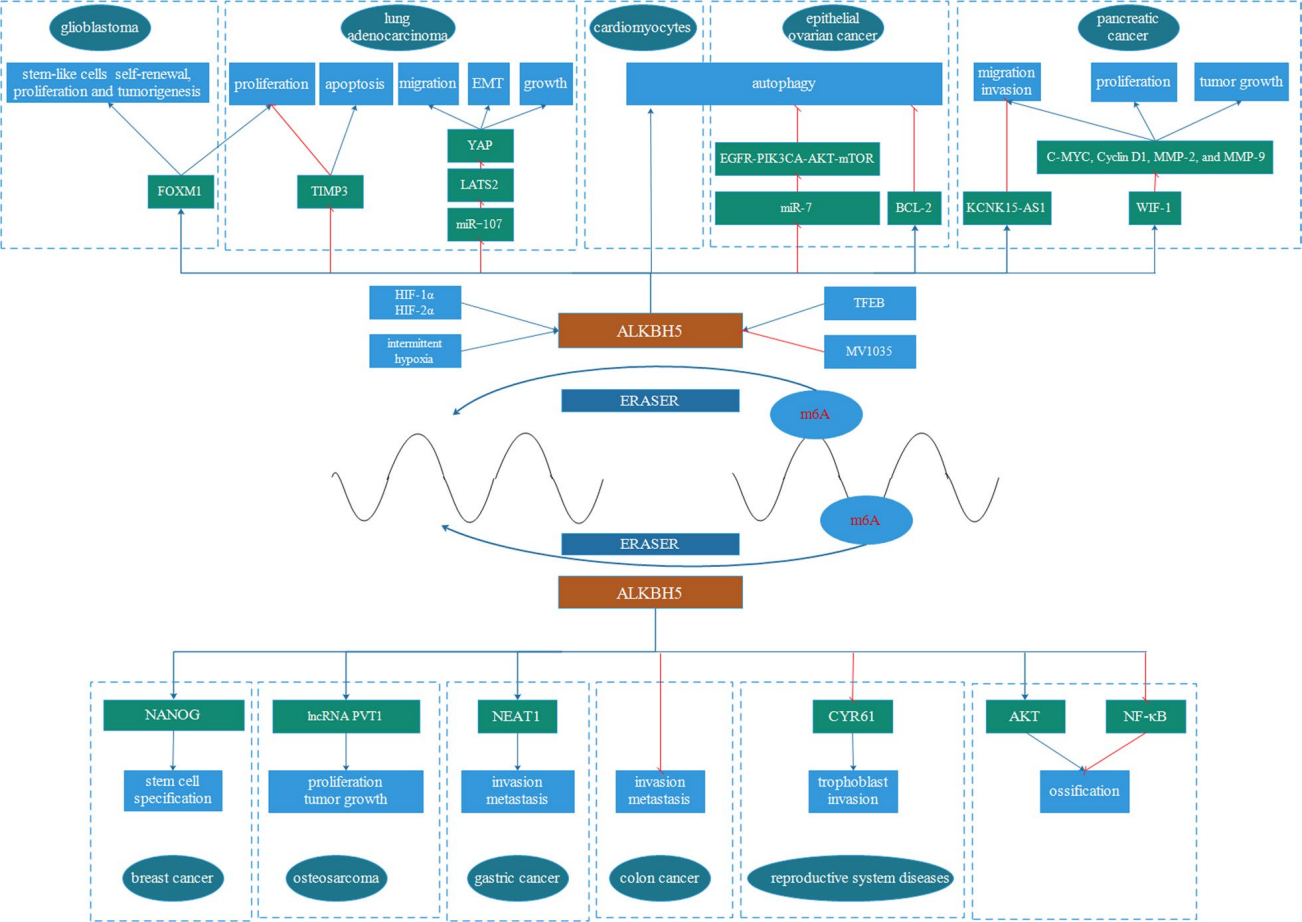
The mechanisms of ALKBH5 are involved in human cancers and non-cancers. ALKBH5 plays an important role in breast cancer, glioblastoma, overian cancer, lung cancer, pancreatic cancer, gastrointestinal cancer and osteosarcoma, by targeting NANOG, FOXM1, EGFR, BCL-2, NEAT1, FOXM1-AS, KCNK15-AS1, PYT1, TIMP3, YAP, KCNK15-AS1 and WIF-1. The underlying mechanisms involve cancer stem cells, autophagy, hypoxia, long non-coding RNA. In addition, ALKBH5 takes a great part in human non-cancers, such as reproductive system diseases and ossification, by targeting AKT and NF-κB. Blue lines indicate promote and red lines indicate inhibit

Human AlkB homolog H5 (ALKBH5), was a famous m6A demethylase (also called m6A eraser), which had spawned significant biological and pharmacological interest among researchers [10]. Recently, the effects of ALKBH5 on many biological processes had been demonstrated, including proliferation [11], invasion [12], metastasis [13], ossification [14] and so on. In addition, ALKBH5 was also involved in kinds of cancers or non-cancers, such as glioblastoma [15], pancreatic cancer [16], colon cancer [17], breast cancer [18], gastric cancer [19], lung cancer [20], ovarian cancer [21], diabetes [22] and reproductive system diseases [23] (Figs. 1, 2).

In the field of cancer research, the underlying mechanisms of ALKBH5 in human cancers were not only unclear but also controversial. Up to date, the expression of ALKBH5 was up-regulated or down-regulated in various cancers, and played an oncogenic or tumor suppressive role in breast cancer, gastric cancer, colon cancer and so on [17, 19, 24]. The potential mechanisms were involved with long non-coding RNAs, miRNAs, mRNA and so on [19, 21, 24]. This kind of communication between ALKBH5 and different RNAs was importantly related with cancer cell proliferation, apoptosis, death, survival, migration, invasion, metastasis and so on [25, 26] (Table 1).

**Table 1:** Expression, clinical significance, and biological function of ALKBH5 in various cancers

Cancer	Expression	Role	Biological Function	Target	References
Lung adenocarcinoma	Upregulated	Oncogene	Proliferation and invasion,	FOXM1	[11]
NSCLC	Upregulated	Oncogene	Proliferation, invasion, migration, and EMT	YAP	[13]
			Proliferation, apoptosis and tumor growth	TIMP3	[20]
Pancreatic cancer	Downregulated	Tumor suppressor	Proliferation, invasion and migration	WIF-1	[16]
			Motility	KCNK15-AS1	[26]
Colon cancer	Downregulated	Tumor suppressor	Invasion and metastasis	–	[17]
Gastric cancer	–	Oncogene	Invasion and metastasis	NEAT1	[19]
Glioblastoma	Upregulated	Oncogene	Migration and invasion	–	[15]
			Proliferation, tumorigenesis	FOXM1	[25]
Epithelial ovarian cancer	Upregulated	Oncogene	Proliferation and invasion	EGFR-PIK3CA-AKT-mTOR, BCL-2	[21]
Clear cell renal cell carcinoma	Downregulated	–	–	–	[45]
Osteosarcoma	–	–	Proliferation and tumor growth	PVT1	[40]
Oral squamous cell carcinoma	–	–	Chemoresistance	FOXM1, NANOG	[46]

In the field of human non-cancer research, ALKBH5 was found to be dysregulated in human reproductive system diseases and osteogenic progression [27, 28]. For example, ALKBH5 acted to influence spermatogenesis and trophoblast invasion, thus affecting human reproductive system diseases [23, 29]. In addition, ALKBH5 also played an important role in the ossification of the ligamentum flavum cells and osteogenic progression via some famous signaling pathways, such as AKT and NF-κB signaling pathway [14, 28].

In this study, we summarized the recent advances made in relation to ALKBH5 dysregulation and its biological role coupled with the underlying mechanisms in various human diseases.

## The structure of ALKBH5

ALKBH5 was one of nine members of the AlkB family, a ferrous iron- and 2-oxoglutarate-dependent nucleic acid oxygenase (NAOX) and had been reported to catalyze the demethylation of m6A in RNA [30]. Zhou et al. [30] and Aik et al. [31] identified the crystal structure of ALKBH5 and revealed conserved residues important for recognition and demethylation mechanisms. Chu et al. [32] and Feng et al. [33] respectively identified the crystal structure of zebrafish ALKBH5 (fALKBH5) and human ALKBH5, which promoted the understanding of the substrate recognition specificity of ALKBH5 and offered a foundation for the development of inhibitors selective for NAOX. In addition, Xu et al. [34] affirmed the m6A binding pocket of ALKBH5 and the key residues related with m6A recognition through mutagenesis and isothermal titration calorimetry binding experiments. Furthermore, m6A served as a ‘conformational marker’, which induced different conformational outcomes in RNAs depending on sequence context and critically impacted its interactions with ALKBH5 [35]. In detail, it was found that the DSBH domain of ALKBH5 indispensablely interacted with ATP domain of DDX3, which was a member of the family of DEAD-box RNA helicases and took a great part in key biological processes such as cell cycle, apoptosis, and RNA metabolism [36]. Purslow et al. [37] further investigated the structure and dynamics of ALKBH5 in solution and offered the first atomic-resolution model of an AlkB protein in its disordered conformational state. There was also a great connection between single-nucleotide polymorphisms (SNPs) on the two RNA demethylases FTO and ALKBH5 [38]. At the same time, he reported the backbone ^1^H, ^15^N, ^13^C chemical shift assignment of a fully active, 26 kDa construct of ALKBH5 [39].

## Regulatory mechanisms of ALKBH5

### ALKBH5 with long non-coding RNA

FOXM1-AS, located on chromosome 12(chr12: 2945982-2968961, GRCh37/hg19), was a nuclear lncRNA that facilitated the communication between ALKBH5 and FOXM1 nascent transcripts. In detail, FOXM1-AS was localized in the same cellular fraction as ALKBH5 and FOXM1 nascent transcripts, and further studies indicated that FOXM1-AS up-regulated FOXM1 expression and played an important role in glioblastoma cells tumorigenesis [25]. Moreover, ALKBH5 demethylated lncRNA KCNK15-AS1, which was downregulated in pancreatic cancer tissues compared with the normal tissues, thus elevating pancreatic cancer motility, including migration and invasion [26]. In addition, ALKBH5 suppressed the degradation of lncRNA plasmacytoma variant translocation 1 (PVT1), which was up-regulated in osteosarcoma tissues and cells, thus promoting the osteosarcoma cell proliferation in vitro and tumor growth in vivo through inhibiting the binding of m6A reader YTHDF2 in PVT1 [40]. ALKBH5 decreased the level of lncRNA nuclear paraspeckle assembly transcript 1 (NEAT1), which was overexpressed in gastric cancer cells and tissue, and took a great part in the invasion and metastasis of gastric cancer [19].

### ALKBH5 with cancer stem cells

It was well-known that cancer stem cells were designated as tumor-initiating cells, which were capable of infinite proliferation through self-renewal, giving rise to progeny that were different from them, and forming a secondary (recurrent or metastatic) tumor [41–43]. ALKBH5 promoted breast cancer stem cell (BCSC) specification and enrichment in the tumor microenvironment though catalyzing the demethylation of an adenosine residue in the 3′-UTR of NANOG mRNA [24]. ALKBH5 expression was also elevated in glioblastoma stem-like cells (GSCs), demethylated FOXM1 nascent transcripts and enhanced the expression of FOXM1, thus enhancing stem cells self-renewal, proliferation and tumorigenesis [25].

### ALKBH5 with autophagy

Another m6A related gene METTL3 relieved autophagic flux in hypoxia/reoxygenation treated cardiomyocytes, however ALKBH5 could reverse this effect [44]. In detail, TFEB activated ALKBH5 transcription through binding to the ALKBH5 promoter. However, the inhibition of METTL3 by TFEB was not dependent on transcriptional repression but rather downregulation of mRNA stability. This negative feedback loop provided insight into the vital link bwtween METTL3-ALKBH5 and autophagy. In addition, the ectopic expression of ALKBH5 inhibited the autophagy of epithelial ovarian cancer cells in vitro and in vivo, partly through activating EGFR-PIK3CA-AKT-mTOR signaling pathway, promoting the stability of BCL-2 mRNA, as well as enhancing the interaction between Bcl-2 and Beclin1 [21]. Moreover, ALKBH5 regulated the EGFR expression, relying on HuR and miR-7.

### ALKBH5 with hypoxia

The expression of ALKBH5 was induced by hypoxia in breast cancer cells depending on hypoxia-inducible factor (HIF)-1α and HIF-2α, and the upregulation of ALKBH5 mediated the enrichment of breast cancer cell in hypoxic tumor microenvironment, through enhancing NANOG expression, therefore advancing tumor formation in vivo [24]. Additionally, TFEB acted to induce the expression of ALKBH5 and controlled the lysosomal-autophagic pathway in hypoxia/re-oxygenation treated cardiomyocytes [44]. Lastly, ALKBH5 was also closely related with the process of intermittent hypoxia in lung adenocarcinoma cells. Mechanistic analysis indicated that m6A demethylase ALKBH5 enhanced the proliferation and invasion of lung adenocarcinoma cells under intermittent hypoxia by down-regulating the level of m6A in FOXM1 mRNA and enhancing the translation efficiency of FOXM1 mRNA, resulting in the overexpression of FOXM1 protein [11].

## ALKBH5 dysregulation in human cancers

In the majority of cancer research, the role of ALKBH5 in human cancers was controversial. It had been found to be upregulated or downregulated, and to play an oncogenic or tumor suppressive role in different kinds of cancers. We will discuss the different roles of ALKBH5 in various cancers thereinafter, including breast cancer, lung cancer, pancreatic cancer, glioma and so on.

### ALKBH5 in breast cancer

Recent researches noted that ALKBH5 up-regulated the expression of NANOG by demethylating m6A and promoted the gathering of breast cancer cells in the tumor microenvironment [18, 24]. NANOG, a pluripotency factor, was necessarily required for primary tumor formation and distant metastasis, because of its povital role in the maintenance and specification of cancer stem cells.

### ALKBH5 in lung cancer

ALKBH5 was also elevated in lung adenocarcinoma cells under intermittent hypoxia, and the knockdown of ALKBH5 inhibited the proliferation and invasion of lung adenocarcinoma cells via reducing the m6A level of FOXM1 [11]. Additionally, ALKBH5 was obviously up-regulated in non-small cell lung cancer (NSCLC) tissues and cell lines, compared with the normal controls, and promoted the malignant biological properties of NSCLC cells via inhibiting the TIMP3 mRNA stability depending on m6A demethylation modification [20]. However, ALKBH5 inhibited tumor growth and metastasis partly through inhibiting the expression and activity of YTHDFs-mediated YAP, and regulating miR-107/LATS2 axis in an HuR-dependent manner [13].

### ALKBH5 in gastrointestinal cancer

ALKBH5 was downregulated in pancreatic cancer cells, and inhibited pancreatic cancer motility [26]. It was found that ALKBH5 sensitized pancreatic ductal adenocarcinoma (PDAC) cells to chemotherapy and ALKBH5 remarkably inhibits pancreatic ductal adenocarcinoma cell proliferation, migration, and invasion, partly by altering the expression of Wnt inhibitory factor 1 (WIF-1), which is mediation of the Wnt pathway [16]. ALKBH5 was downregulated in colon cancer tissues, and the overexpression of ALKBH5 greatly reatrained colon cancer cells invasion in vitro and metastasis in vivo [17]. Besides, ALKBH5 participated in the carcinogenicity of gastric cancer by affecting the methylation of NEAT1 [19].

### ALKBH5 in other cancers

Regarding glioblastoma, ALKBH5 provided a deep insight into critical roles of m6A methylation though enhancing the expression of FOXM1, and promoted tumorigenesis in glioblastoma [25]. And the imidazobenzoxazin-5-thione MV1035 inhibited glioblastoma cell line migration and invasion via inhibiting ALKBH5 [15].

As for other cancers, ALKBH5 was significantly downregulated in clear cell renal cell carcinoma (ccRCC) compared to normal tissue [45]. It was also affirmed that ALKBH5 took a great part in contributing to osteosarcoma tumorigenesis [40]. ALKBH5, directly regulated by human RNA helicase DDX3, decreased m6A methylation in FOXM1 and NANOG nascent transcript and contributed to chemoresistance of oral squamous cell carcinoma(OSCC) [46]. Besides, ALKBH5 was upregulated in epithelial ovarian cancer tissue compared with the normal ovarian tissues, and enhanced the proliferation and invasion in vitro and in vivo, not only through activating EGFR-PIK3CA-AKT-mTOR signaling pathway, but also through strengthening the stability of BCL-2 mRNA and the interaction between Bcl-2 and Beclin1 [21].

### ALKBH5 in cancer prognosis

Notably, decreased ALKBH5 was closely connected with a shortened overall and cancer-specific survival following nephrectomy in ccRCC [45]. What’s more, decreased expression of ALKBH5 was closely associated with metastasis and American Joint Committee on Cancer (AJCC) stage and acted as an independent prognostic indicator in colon cancer patients [17]. Tang, et al. [16] found that low level of ALKBH5 predicted poor clinical outcome in pancreatic ductal adenocarcinoma, phenochromocytoma, paraganglioma, stomach adenocarcinoma and uterine corpus endometrial carcinoma. In detail, the expression of ALKBH5 was also connected with specific clinicopathological features such as TNM staging, tumor size, lymph node metastasis, and distant metastasis. Nontheless, such association could not be observed in breast cancer and head-neck squamous cell carcinoma. Also, Cho, et al. [47] analyzed that ALKBH5 expression was positively associated with overall survival and was a new independent prognostic marker for pancreatic cancer though a retrospective multicohort study.

On the contrary, low level of ALKBH5 was associated with better patients’ survival in bladder cancer [16]. Besides, the up-regulated ALKBH5 predicted poor survival in glioblastoma patients [25] and NSLCL patients [20].

The above evidences suggested that the role of ALKBH5 in the prognosis of cancers might be context-dependent.

## ALKBH5 dysregulation in human non-cancer

ALKBH5 was also connected with human non-cancer, such as reproductive system diseases and the process of ossification.

### ALKBH5 in reproductive system diseases

ALKBH5 was the main eraser of the m6A modification in germ cell tumors [48]. ALKBH5 functioned to oxidatively reverse m6A in vitro and vivo, and ALKBH5-deficient male mice had increased m6A in mRNA and were connected with compromised spermatogenesis through affecting mRNA export and RNA metabolism [29]. Inactivation of ALKBH5 lead to male infertility in mice partly though aberrant splicing and production of shorter transcripts [23]. Moreover, ALKBH5 expression was specifically unregulated in placental villous tissue from recurrent miscarriage patients, and overexpression of ALKBH5 inhibited trophoblast invasion by villous explant culture experiments, through decreasing the half-life of CYR61 mRNA and inhibiting steady-state CYR61 mRNA expression levels [12].

However, it was reported that reduced semen quality exhibited no correlation with genetic variants in the genes coding for the messenger RNA methylation erasers ALKBH5 [27].

### ALKBH5 in ossification

ALKBH5 was increased in ossification of the ligamentum flavum (OLF) tissues, and promoted the ossification of the ligamentum flavum cells through BMP2 demethylation and activating AKT signaling pathway [14]. The METTL3-mediated m6A methylation was found to be dynamically reversed by the demethylase ALKBH5, which acted to reverse the suppression of METTL3 in osteogenic progression, partly though regulating expression of MYD88 and facilitating the activation of NF-κB which was a well-known repressor of osteogenesis [28].

## Conclusion

The data described in this review suggested the controversial expression patterns and functions of ALKBH5. The mechanisms of ALKBH5 were involved in human cancers and non-cancers. On the one hand, ALKBH5 was up-regulated in kinds of cancer tissues and cell lines, such as breast cancer, lung cancer and epithelial ovarian cancer, and played an oncogenic role in tumor progression. On the other hand, other researchers indicated the opposite conclusions about the expression level and the role of ALKBH5 in pancreatic cancer, colon cancer and clear cell renal cell carcinoma. ALKBH5 regulated cancer cell proliferation, migration, invasion, tumor progression, metastasis, tumorigenesis and chemoresistance through modulating m6A methylation. These effects were orchestrated through multiple mechanisms, such as long non-coding RNAs, cancer stem cells self-renewal and autophagy. ALKBH5 could demethylate lncRNAs, enhance cancer cells self-renewal or regulate autophagy in cancers. Furthermore, ALKBH5 played a great role in human non-cancers, such as reproductive system diseases. Additionally, ALKBH5 had great potential for clinical application by acting as a new prognostic target. However, further researches are still needed to illuminate the exact details of ALKBH5 expression and its mechanisms in human cancers and non-cancers.

## Data Availability

Not applicable.
